# Glioblastoma and Methionine Addiction

**DOI:** 10.3390/ijms23137156

**Published:** 2022-06-28

**Authors:** Mark L. Sowers, Lawrence C. Sowers

**Affiliations:** 1 Department of Pharmacology and Toxicology, University of Texas Medical Branch, 301 University Boulevard, Galveston, TX 77555, USA; mlsowers@utmb.edu; 2 MD-PhD Combined Degree Program, University of Texas Medical Branch, 301 University Boulevard, Galveston, TX 77555, USA; 3 Department of Internal Medicine, University of Texas Medical Branch, 301 University Boulevard, Galveston, TX 77555, USA

**Keywords:** glioblastoma, methionine, metabolism, tumor microenvironment, epigenetics, therapeutic development

## Abstract

Glioblastoma is a fatal brain tumor with a bleak prognosis. The use of chemotherapy, primarily the alkylating agent temozolomide, coupled with radiation and surgical resection, has provided some benefit. Despite this multipronged approach, average patient survival rarely extends beyond 18 months. Challenges to glioblastoma treatment include the identification of functional pharmacologic targets as well as identifying drugs that can cross the blood-brain barrier. To address these challenges, current research efforts are examining metabolic differences between normal and tumor cells that could be targeted. Among the metabolic differences examined to date, the apparent addiction to exogenous methionine by glioblastoma tumors is a critical factor that is not well understood and may serve as an effective therapeutic target. Others have proposed this property could be exploited by methionine dietary restriction or other approaches to reduce methionine availability. However, methionine links the tumor microenvironment with cell metabolism, epigenetic regulation, and even mitosis. Therefore methionine depletion could result in complex and potentially undesirable responses, such as aneuploidy and the aberrant expression of genes that drive tumor progression. If methionine manipulation is to be a therapeutic strategy for glioblastoma patients, it is essential that we enhance our understanding of the role of methionine in the tumor microenvironment.

## 1. Introduction

### 1.1. Methionine Addiction Is a Metabolic Vulnerability in Cancer

Studies in the 1970s [1,2,3] examined the requirements of both normal and cancer cells in culture for amino acids and metabolites including methionine. Normal cells were shown to propagate in vitro in the presence of homocysteine, the metabolic precursor to methionine, while many cancer cell lines could not. The observation of methionine dependence in many, but not all human cancer cell lines [4] suggested that this biochemical difference between normal and malignant cells could be exploited therapeutically.

Extending the cell culture work to primary tumors, Guo et al. demonstrated methionine dependence in 5 of 21 primary human malignant tumors from multiple tissues [5]. In 1994, Fiskerstrand attributed the methionine dependence of glioblastoma in culture to low homocysteine remethylation, which resulted from low methylcobalamin (vitamin B12) levels [6]. In 2017, a proteomic study with U87 cells grown under hypoxic conditions revealed significantly reduced expression of the TCN2 transporter that delivers vitamin B12 to tumor cells, consistent with the Fiskerstrand results [7]. Recently, Wang et al. showed that tumor-initiating cells (TIC) within heterogenous tumors have an elevated methionine cycle driven by increased MAT2A, which converts methionine to S-Adenosyl methionine (SAM) [8]. The upregulation of the methionine cycle could cause methionine consumption to exceed the rate of production. It is likely that multiple factors in tumor cells combine to create a dependence on exogenous methionine, and the relative contribution of these factors could differ from one cell to another and depend heavily on conditions in the tumor microenvironment. However, recapitulating the tumor microenvironment in a laboratory setting is an ongoing challenge of critical importance.

### 1.2. Traditional Methods for In Vitro Cell Culture Do Not Reflect the Tumor Microenvironment

Metabolic differences between normal and tumor cells have the potential for serving as selective targets that disadvantage tumor cells. However, the conditions under which cells are routinely propagated in the laboratory can be very different from those in the tumor microenvironment. This is a fundamental challenge with previous, as well as many ongoing studies, of cell metabolism.

Traditional culturing methods have not changed substantially over the past 80 years since the development of Eagle’s minimal essential medium (EMEM) and Dulbecco’s Modified Eagle’s Medium (DMEM). The primary advantage of these culture conditions is that they are effective in promoting cell propagation. Understanding and subsequently recapitulating the tumor microenvironment will be a key factor in discovering novel metabolic drug targets.

**Figure 1:**
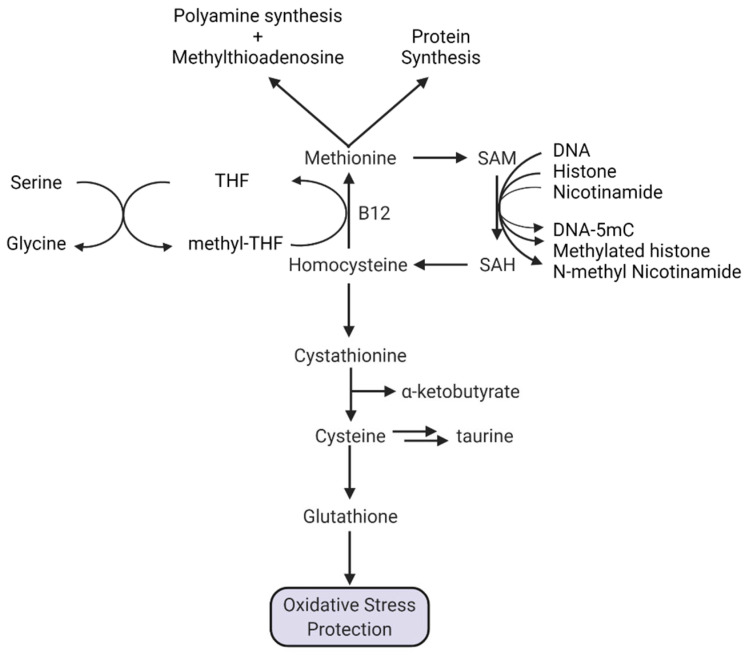
Methionine metabolic pathways.

The abundance of nutrients found in standard culture media could profoundly alter the metabolism of the tumor cells [9]. For example, standard DMEM media contains ~400 μM methionine whereas serum methionine concentration is ~130.6 ± 92.9 μM and in CSF it is ~13.9 ± 2.6 μM [10]. Others have also reported lower levels around ~30–40 μM in serum and ~3 μM in CSF [11,12]. Furthermore, culturing cells in 20% O_2_, in contrast to a hypoxic tumor microenvironment, could require shunting of the methionine precursor, homocysteine, towards glutathione synthesis to counteract increased levels of reactive oxygen species (Figure 1). Decreased methionine synthesis could therefore result in part from decreased homocysteine availability [13].

Another critical component that is in excess in typicalmedia is glucose. Glucose consumption has been a major focus of metabolic differences between normal and cancer. It has long been appreciated that cancer cells frequently consume vast amounts of glucose and produce lactate; this is called the ‘Warburg effect.’ Interestingly, DMEM media used to culture cancer cells typically contains 25 mM glucose while serum concentrations in humans are estimated to be ~3.5–7 mM and ~4 mM in CSF [14]. These supraphysiologic, but nonetheless historical, cell culture conditions may affect our understanding of cancer metabolism in its native microenvironment.

Increasing attention is being focused on media composition in cell culture so that it more closely reflects physiological conditions [12,15,16,17], and in particular, the tumor microenvironment. Cantor et al. demonstrated that the metabolic landscape of cells is extensively altered when comparing cells grown in RPMI1640 versus their human plasma-like medium (HPLM) [12]. Similarly, Vande Voorde et al. argued that a serum-like amino acid composition and the addition of selenite better approximates the metabolic profile of human breast tumors, in contrast to DMEM which introduces metabolic artifacts [16]. Others studying GBM found that when compared to previous studies using DMEM, their more physiologic medium demonstrated that GBM cells likely produce net glutamine [18] rather than consume it as a fuel source as previous reports had indicated [19].

Efforts are underway in many laboratories to identify additional factors that should be examined including the extracellular matrix composition and oxygen tension. With respect to metabolic manipulation of tumor cell growth, attention to media composition will be critical for recapitulating the tumor microenvironment.

The growing interest in precision medicine is built upon assumptions that cancer cell-specific properties, including genetic and epigenetic perturbations, determine how tumor cells will respond to specific therapies. If alterations in cellular metabolism are being targeted, nutrient availability will impact response to those drugs. Therefore, the proper formulation of cell culture conditions is essential to determine how the tumor nutrient environment might influence drug response in patients. Recent formulations of culture media based upon the composition of human serum represent a significant improvement [12,16,18].

### 1.3. The Accumulation of ^11^C-Methionine by Glioblastoma Tumor Cells Is Diagnostic and Reveals Important Aspects of Tumor Biology

While numerous metabolic differences between cancer and normal tissues have been appreciated, recent studies using more physiologic media are challenging our current understanding. Consequently, it is unclear which metabolic defects identified in cancer cells are more likely artifacts of historical cell culture conditions. However, some of the most compelling evidence for the requirement of glioblastoma for exogenous methionine is from ^11^C-methionine positron emission tomography (PET) imaging which highlights glioblastoma tumors in patients [20,21,22]. Methionine transits the blood-brain barrier (BBB) and enters tumor cells using the LAT1 transporter. In 2005, Nawashiro et al. demonstrated high expression of the L-type amino acid transporter 1 (LAT1) in infiltrating glioma cells from primary glioma tissues [23]. Increased transport of methionine into glioma cells suggests a mechanism for responding to defects in intracellular methionine synthesis. More recently, new LAT1 PET probes have shown low accumulation in normal brain tissue, but high accumulation in grade IV glioma like ^11^C-MET [24].

The anatomic resolution possible with ^11^C-MET PET can reveal important aspects of gliomagenesis. Van Dijken et al. recently showed that aggressive tumors that grow near brain ventricles also accumulate more ^11^C-MET [25]. The subventricular zone (SVZ) is an important neural stem cell-containing niche. It is believed that gliomas can arise from this cell population [26,27]. Glioma tumors in contact with the ventricles are often more multifocal and can recur distally from the primary tumor. Therapeutic resistance, tumor aggressiveness, and shorter patient survival are all associated with tumors in contact with ventricles [28,29,30,31,32].

While earlier studies of glioblastoma highlighted the capacity of PET to demarcate the tumor, recent efforts have used PET in conjunction with other modalities. While ^11^C-MET PET can distinguish tumor tissue from inflammatory tissue better than ^18^F-FDG, ^11^C-MET PET requires special facilities including a cyclotron. A fluorescent marker that can be used intraoperatively to facilitate tumor resection is based upon the metabolism of 5-ALA to a naturally fluorescent molecule, protoporphyrin IX (PpIX) [33,34]. Prior studies have shown that glioma cells accumulate PpIX because they metabolize it to heme more slowly than normal cells, or export it less efficiently. Shimizu et al. studied a series of glioma patients and established that 5-ALA-mediated tumor fluorescence, measured ex vivo, correlated with ^11^C-MET labeling in vivo [35]. While it is not as yet known mechanistically if ^11^C-MET and PpIX accumulation are metabolically linked, they both occur within tumor cells in the same anatomic location.

Gadolinium (Gd) enhancement and magnetic resonance imaging (MRI) is another traditional approach for imaging glioblastoma. However, tumor volumes indicated by Gd enhancement are generally smaller than volumes indicated by ^11^C-MET PET, particularly for highly infiltrative tumors. Inoue et al. demonstrated that glioma stem cells (GSC) often exist at the periphery of tumors that do not show Gd enhancement [36]. A series of tumors were evaluated simultaneously by Gd-enhancing MRI and ^11^C-MET PET and classified as having high or low invasiveness on the basis of overlapping tumor volumes measured by each method. RNA was isolated from both core and peripheral areas of a series of resected tumors. The levels of transcripts for two genes involved in energy metabolism, lactate dehydrogenase A (LDH-A) and pyruvate dehydrogenase (PDH) as well as the stem cell marker CD44 [37] were measured. In the presence of oxygen, pyruvate can be converted to acetyl-CoA by PDH and then enter the TCA cycle. In hypoxic regions of the tumor, including the tumor periphery, pyruvate is converted to lactate by LDH-A allowing continuous glycolysis. Analysis of mRNA by qRT-PCR showed that LDH-A was expressed at significantly higher levels on the periphery of highly invasive tumors whereas PDH was expressed at higher levels in the less invasive tumors [36]. In vitro studies with a series of GSC stem cells showed that expression of CD44 and LDH-A mRNA correlated with the invasiveness of the cell lines. The authors concluded that highly invasive GSCs, which consume a significant amount of methionine but little oxygen, exist at the periphery of glioblastoma tumors [36].

The accumulation of ^11^C-MET can be used to estimate prognosis in patients with grade II to grade IV astrocytomas [38]. The greater the overall accumulation of ^11^C-MET, the shorter the patient survival. Maeda et al. showed that fractal analysis of ^11^C-MET PET in patients with newly diagnosed GBM could differentiate low-grade glioma and glioblastoma as well as indicated the IDH1 mutation status [39]. The fractal dimension measures the pattern of the radioactivity distribution in the region of interest. The uptake of ^11^C-MET is lower in IDH-mutant tumors, which tend to be less aggressive.

Collectively, in vitro data, coupled with more direct studies with human tumor tissue suggest that glioblastomas, as with many other human tumors, have either defective methionine synthesis, or have an unusual demand for exogenous methionine, or some combination of both. The accumulation of methionine measured by ^11^C-MET-PET, in combination with other modalities, can be useful for distinguishing tumor recurrence from radiation necrosis, revealing the anatomic location of GSCs, and providing additional characterization that can benefit the evaluation of patient prognosis.

### 1.4. Is Dietary Methionine Restriction a Potential Treatment Strategy?

The apparent dependence of many human tumors on exogenous methionine has led to proposals that methionine restriction or depletion might provide a way to slow or cure human tumors. In 1959, Sugimura et al. showed that a diet devoid of methionine, leucine, or valine reduced the growth of Walker tumors implanted into the flanks of Sprague-Dawley rats [40]. Subsequent animal studies have shown additional health benefits of reduced methionine consumption including decreased adiposity, increased insulin sensitivity, decreased inflammation and oxidative stress as well as extended lifespan. These results suggest that dietary methionine restriction could benefit cancer patients [41,42,43,44].

In human studies, dietary methionine depletion has been synergistic with 5-fluorouracil (5FU) in patients with advanced gastric cancer [45]. In a phase 1 study involving eight human subjects [46], it was shown that plasma methionine levels could be reduced from 21 ± 7.3 μM to 9 ± 4 μM within two weeks on a methionine-free diet. In 2009, Thivat et al. examined the combination of the chloroethylnitrosurea, cystemustine, and a methionine-free diet in 22 patients with melanoma and glioblastoma [47]. While no objective response was observed, two patients had long-duration stabilization.

In addition to dietary restriction, plasma methionine levels can be further reduced in humans using recombinant methioninase with no clinical toxicity [48]. Encapsulated methionine γ-lyase [49] reduced plasma methionine levels to 40% of pretreatment levels and slowed the growth of implanted glioblastoma xenografts in nude mice. Evidence suggests that methionine dietary restriction, coupled with cytotoxic chemotherapy might slow the progression of some human tumors.

### 1.5. Why Does Glioblastoma Need Exogenous Methionine?

While methionine restriction is compelling as a potential treatment strategy, further studies are required to understand the mechanistic basis of methionine addiction in glioblastoma. Such studies should seek to understand the factors that could result in deficits in homocysteine remethylation to methionine (Figure 1). Further, understanding the apparent increased demand of tumor cells for exogenous methionine might illuminate more effective pharmacological targets.

#### 1.5.1. Methionine and Protein Synthesis

Methionine is an amino acid found in most proteins, and protein synthesis likely consumes a considerable portion of methionine. In eukaryotes, methionine is the first amino acid at the AUG start codon for the majority of proteins. Occasionally, alternative start sites utilizing other codons might occur when methionine is limited. Some investigators have suggested that under low methionine, the transcription of many proteins will be stopped in favor of the transcription of an alternative set of proteins creating an altered proteomic program in response [50]. This could be an unintended consequence of methionine depletion.

The methionine on the amino terminus of newly synthesized proteins is often removed enzymatically. Removal of the terminal methionine can be necessary for protein stability, activation and cellular localization. Methionine aminopeptidase (MetAP2) removes the terminal methionine from newly synthesized proteins, and it is overexpressed at both the mRNA and protein levels in high grade gliomas. Lin and coworkers have shown that the knockdown of MetAP2 decreased cell proliferation and decreased angiogenesis. With respect to methionine consumption, high MetAP2 activity would facilitate the recycling of methionine, increasing methionine availability [51].

#### 1.5.2. Methionine and Polyamine Synthesis

Methionine is also used for polyamine synthesis. The polyamines spermine, spermidine and putrescine are cationic amines critical for cellular growth, proliferation, and differentiation. During the conversion of putrescine to spermidine and spermidine to spermine, carbon atoms derived from the methionine backbone of SAM are used, converting SAM to 5′-deoxy-5′-methylthioadenosine (MTA). Polyamine levels are higher in cancer cells [52] perhaps explaining in part increased utilization of methionine in high-grade gliomas.

The reutilization or salvaging of MTA, generating adenine and methionine, requires methylthioadenosine phosphorylase (MTAP). The MTAP gene is found on chromosome 9p21, and regions of this chromosome are deleted in almost half of GBMs [53]. MTAP deletion would prevent the recycling of methionine, reduce methionine pools and increase intracellular MTA. MTA is a strong inhibitor of spermine synthesis [54]. MTA is also an inhibitor of MAT2A which would reduce SAM levels [55]. Another enzyme inhibited by MTA is arginine methyltransferase (PRMT5). PRMT5 acts in multiple signaling pathways by converting arginine residues in proteins to symmetrically dimethylated residues. The inhibitory activities of MTA led to suggestions that the inhibition of PRMT5 by small molecules could be preferentially toxic to MTAP-deficient cells [56,57].

More recent studies suggest that MTA accumulation in MTAP deficient glioblastoma might not be as lethal as anticipated. In a series of glioblastoma cell lines, MTAP-deficient cells grown in 100 μM methionine resulted in the accumulation of high levels of MTA [58]. However, when the methionine concentration in the media was reduced to 3 μM, MTA levels were significantly diminished to those found in MTAP-competent cells. These results highlight the need to consider dietary nutrient composition in experimental cell culture studies.

In a similar manner, others have considered the potential role of the tumor microenvironment in therapy directed against MTAP-deficient cells [59]. Glioblastoma tumors are heterogenous, containing distinct tumor cell types as well as normal cells including neurons, astrocytes, microglia, macrophages, neutrophils, lymphocytes, and endothelial cells. While higher levels of MTA were found within cultured MTAP-deficient cells, substantially greater levels were found in the extracellular media. In primary resected MTAP-deficient GBM tumors, no significant increases in MTA levels were observed. Previously, Locasale et al. measured MTA levels in the CSF from GBM and normal brains [60]. Surprisingly, no significantly elevated levels of MTA were found in any of the patients even though ~50% of GBMs are MTAP-deficient. These results suggest that MTA exported from MTAP-deficient cells can be metabolized by MTAP-proficient cells in the tumor microenvironment.

#### 1.5.3. Methionine, SAM, Transmethylation, and NAD+

**Figure 2:**
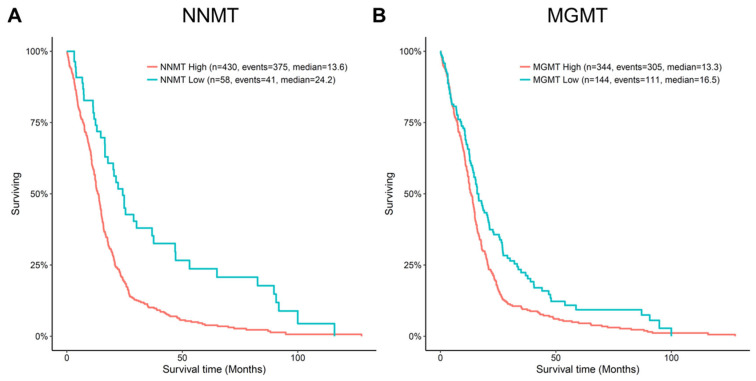
Kaplan Meier curves of NNMT and MGMT microarray-based expression. High expression of either gene is associated with poor survival with a Wilcoxon p value of 1 × 10^−4^ and 7.1 × 10^−3^ for NNMT and MGMT, respectively. Data was obtained and visualized using the TCGA-GBM study Agilent-4502A microarray data. Optimal cutoff points for survival curves were determined using the survminer package, −1.93 and −1.39 for NNMT and MGMT, respectively. Visualization and statistics of this data were prepared using http://gliovis.bioinfo.cnio.es/ (accessed on 1 May 2022) [64].

Methionine is also a methyl group donor for numerous S-adenosylmethionine-dependent enzymatic methylation reactions. Small molecules, including melatonin, epinephrine, and N-methylnicotinamide are the products of SAM-dependent methylation (Figure 1). Among these pathways, the SAM-dependent methylation of nicotinamide by nicotinamide-N-methyl transferase (NNMT) is of high significance in glioblastoma. NNMT is commonly overexpressed in human glioblastoma [61,62,63] and levels correlate with aggressiveness. High NNMT transcriptomic expression is negatively associated with patient survival (Figure 2A) [64], suggesting that methionine consumption by NNMT might be an important factor in methionine dependence, but why is NNMT important in tumor cells?

**Figure 3:**
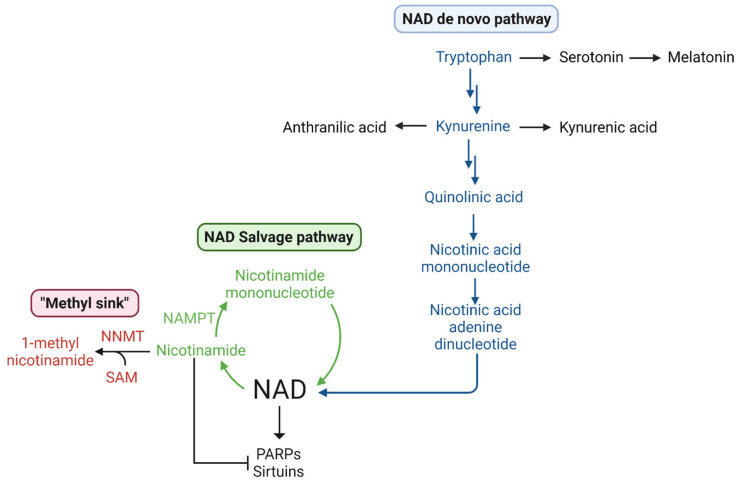
NAD Synthesis by *de novo* and salvage pathways.

NAD+ is central to metabolism, signal transduction, and bioenergetics in all cells. Two major families of signaling pathways, sirtuins and poly-ADPribose polymerases (PARP), consume NAD+. Sirtuin-mediated signaling operates through protein deacetylation and NAD+ hydrolysis. PARPs are important in cellular processes including DNA repair where they act as damage sensors forming a polymer of ADP-ribose connected to proteins and DNA [65,66]. Both sirtuins and PARPs consume NAD+ and generate nicotinamide (Nam). Nam is a feedback inhibitor of both sirtuins and PARPs. Nam can be metabolized by nicotinamide phosphoribosyltransferase (NAMPT) to nicotinamide mononucleotide (NMN) and then recycled to NAD+ [67] (Figure 3). Alternatively, Nam can be methylated to N-methylnicotinamide by NNMT and excreted [68], revealing an intersection of methionine metabolism and cellular NAD+ levels.

Dysregulated NAD+ metabolism and loss of NAD+ is a hallmark of aging and many neurodegenerative diseases [68,69]. The existence of NNMT is therefore paradoxical, as it diverts Nam from recycling by NAMPT and it consumes SAM. However, in response to DNA damage by chemotherapy agents including TMZ and radiation, nicotinamide (Nam) must be rapidly metabolized to allow unabated PARP-mediated DNA repair. The levels of both NAMPT and NNMT are increased in high-grade gliomas, and they must compete for Nam. NAMPT, however, has a very high affinity for Nam (*K_m_* 5 nM) whereas NNMT has a lower affinity (*K_m_* 400 μM) [70]. This difference in substrate affinity means that Nam will be recycled at low levels and only diverted and excreted at high levels. The essential role of NAMPT in maintaining NAD+ levels has led to NAMPT inhibitors that can trigger apoptosis in glioma stem-like cells [71].

In addition to the recycling of Nam by NAMPT, NAD+ can be synthesized by two pathways [69]. Dietary nicotinic acid (niacin, vitamin B3) can be converted to NAD+ in three steps. Alternatively, tryptophan can be metabolized by the kynurenine pathway to kynurenine and then quinolinic acid which can be converted to NAD+. The kynurenine pathway is hyperactive in glioblastoma [72,73] and it supports tumor development and progression [74,75], although perhaps not by engaging the aryl hydrocarbon receptor, AHR [76].

Increased expression of NAMPT and NNMT would allow glioblastoma cells to rapidly respond to sirtuin signaling and DNA damage and may in part explain chemotherapy and radiation resistance in glioblastoma stem-like cells. The consumption of SAM required by this pathway might also explain the excess demand for methionine by glioblastoma cells.

#### 1.5.4. A Special Role for Methionine in DNA Methylation

In addition to small molecules, biological macromolecules including DNA, RNA, and proteins are also methylated in SAM-dependent pathways. Cytosine in CpG dinucleotides in DNA can be methylated enzymatically, forming 5-methylcytosine (5mC), using SAM as the methyl donor (Figure 1). The location of 5mC bases along the DNA strands comprises a methylation pattern within a cell. The maintenance methyltransferase DNMT1, follows closely behind the DNA replication fork allowing methylation patterns to be copied to progeny cells [77]. Alterations in DNA cytosine methylation patterns have long been recognized in human cancer cells. Regional hypermethylation, coupled with global hypomethylation is a common finding in human cancer [78]. Perturbations in DNA methylation are involved in cancer etiology in three distinct ways: (1) the methylation status of gene promoter regions can regulate transcription, (2) the deamination of 5mC to T at CpG dinucleotides within gene coding regions can result in transition mutations that disable tumor suppressor proteins, and (3) the loss of methylation in repetitive DNA regions can result in aneuploidy.

The transcription of specific genes in eukaryotes is controlled by multiple mechanisms. The methylation of specific cytosine bases within gene promoter regions, coupled with the modification of associated histone proteins, comprises the epigenetic control of gene transcription. In glioblastoma, the aberrant hypermethylation of several genes, resulting in transcriptional silencing, is associated with poor patient prognosis [79]. Alternatively, the promoters of transforming genes can become hypomethylated, resulting in aberrantly increased gene expression.

Cytosine methylation is not limited to gene promoter regions. Most CpG dinucleotides within gene coding regions are also methylated [80]. Methylation within these coding regions is not correlated with decreased transcription, as is true for methylation in gene promoters. Rather, methylation of CpG dinucleotides within gene coding regions promotes the binding of proteins with methyl-binding domains (MBD) which function as chromatin organizers. Mutations in one MBD protein, methyl CpG binding protein 2 (MeCP2) underlie the development of Rett Syndrome which is characterized by severe neurological impairment [81]. Within coding regions, C to T transition mutations occur with hotspot frequency at CpG dinucleotides in glioblastoma due to the hydrolytic deamination of 5mC to T [82,83,84].

A third way in which methylation is involved in cancer etiology is the methylation of repetitive DNA sequences. Although the exact mechanisms are currently unknown, loss of methylation in repetitive sequences is associated with ineffective chromosome segregation and aneuploidy [85,86].

### 1.6. Potential Consequences of Methionine Restriction in Glioblastoma Patients

While dietary restriction and enzymatic depletion of methionine might benefit some cancer patients, this strategy might be more difficult to implement with brain tumors. Locasale et al. examined metabolites in CSF from glioma patients and controls [60]. Surprisingly, despite the accumulation of methionine by tumor cells revealed by ^11^C-MET PET discussed above, the level of methionine in the CSF of glioma patients was more than twice the level in noncancer controls. Nicotinamide levels were lower in glioma patient CSF, perhaps due to increase NAMPT and NNMT activities.

LAT1-PET probes also accumulate in glioblastoma [24,87]. Previously Haining and coworkers showed that LAT1 overexpression is associated with glioma grade and that LAT1 was expressed in both tumor cells and vascular endothelial cells [88]. LAT1 expression was associated strongly with microvascular density suggesting it might play a role in the neovascularization of gliomas. LAT1 transports methionine into glioma cells, and it also transports methionine across the blood-brain barrier. Overexpression of LAT1 in gliomas might in part explain elevated CSF methionine levels. 

Shen et al. compared plasma metabolite levels with 2-year overall survival of glioblastoma patients [89]. Higher levels of methionine and arginine were associated with longer survival whereas higher kynurenate was associated with shorter survival. Perhaps increasing tumor burden diminishes plasma methionine. Higher kynurenate levels indicate increased *de novo* NAD+ synthesis, perhaps in response to NNMT depletion of Nam and SAM. Reducing CSF methionine levels as a treatment approach to methionine-addicted tumor cells might be more difficult in the context of gliomas due to the role of LAT1 in the delivery of methionine to the tumor microenvironment. Potential treatment strategies may require simultaneous methionine depletion and antagonism of the LAT1 transporter.

Even if methionine levels in the CSF could be significantly reduced, depletion of exogenous methionine could potentially alter the epigenetic landscape and perturb gene transcription in tumor cells in adverse ways. The most aggressive molecular subtype of glioblastoma is the mesenchymal subtype [90]. The mesenchymal subtype is associated with tumor recurrence as well as chemo and radio-resistance [91]. The ANXA2 gene product is a positive regulator of the mesenchymal subtype, and loss of DNA methylation in the promoter of the ANXA2 gene can drive mesenchymal transformation [92]. Loss of methylation in promoters of other genes, such as uridine phosphorylase (UPP1), is also associated with decreased survival [79].

The primary chemotherapy drug for glioblastoma, temozolomide (TMZ) is an alkylating agent causing DNA base methylation. However, TMZ methylation is not at the C5 position of cytosine, but primarily at the 6-oxygen of guanine (O6G) and 3-nitrogen of adenine (N3A). The presence of O6G results in stalled DNA replication. The DNA repair enzyme, methylguanine methyltransferase (MGMT) can repair alkylation damage caused by TMZ. Loss of cytosine methylation in the promoter of the MGMT gene results in increased MGMT expression, reduced sensitivity to TMZ, and potentially decreased patient survival [93,94].

In older patients with primary glioblastoma, decreased exogenous methionine, coupled with decreased methylation of the MGMT promoter, could result in MGMT expression, increased repair of TMZ-alkylation damage, and decreased patient survival. However, increased MGMT transcriptomic expression is not associated with a difference in survival for low-grade gliomas (Figure 2B). This apparent discrepancy in tumor type is as yet unexplained.

Regional hypermethylation of gene promoters is associated with decreased gene transcription in human cancer. However, the loss of global cytosine methylation is a hallmark of advanced human cancers [78]. Cytosine methylation within repetitive DNA sequences facilitates proper chromosomal segregation during mitosis, and loss of methylation could induce aneuploidy. Levels of 5mC in human cancer cell lines are uniformly lower than those in normal tissues [95]. Transient silencing or gene disruption of DNMT1 in HCT116 cells results in aneuploidy [96,97]. Treatment with the demethylating drug 5-aza-2′-deoxcytidine similarly causes genetic instability and aneuploidy [85]. The mechanism by which loss of global methylation results in aneuploidy is not completely understood. However, cytosine methylation is important for the DNA binding of proteins containing a methyl-binding domain (MBD) such as MeCP2, and MeCP2 is known to be important for proper mitotic spindle organization [98]. Consistent with this mechanism, the loss of another methyl-binding protein, ZBTB4, can result in increased aneuploidy and tumorigenesis [99]. Depletion of methionine, resulting in global demethylation, could drive aneuploidy and tumor progression.

In studies with human gliomas, loss of 5mC was associated with increasing tumor grade [100]. In animal models of glioblastoma, folate supplementation, as opposed to methionine depletion, is reported to limit glioma aggressiveness by remethylation of DNA repeat elements [100,101]. Studies with patient-derived glioblastoma stem cells [102] showed that methionine depletion inhibited tumor growth and reduced the expression of DNMT1 and DNMT3A. However, methionine restriction also upregulated NNMT expression, promoted DNA hypomethylation, and promoted a proneural-to-mesenchymal transition. Therefore, methionine depletion could have undesirable effects through epigenetic changes that rewire gene expression towards a more aggressive phenotype.

## 2. Conclusions

Methionine is a critical amino acid involved in multiple biochemical pathways. A decreased capacity to convert homocysteine to methionine, coupled with increased utilization of exogenous methionine, makes cancer cells more dependent upon methionine for proliferation. However, methionine also links the tumor microenvironment with epigenetic reprogramming. Methionine depletion could then lead to reduced expression of DNMT1, reduced SAM levels, and DNA hypomethylation. Hypomethylation within some promoters could induce expression of genes associated with decreased patient survival, while loss of global methylation could promote aneuploidy and promote proneural-to-mesenchymal transition of glioblastoma stem cells. Further studies will be required to sort out the mechanistic details and such studies may provide novel pharmacological targets.

## Data Availability

Data is contained within the article.

## References

[1] Chello P.L., Bertino J.R. (1973). Dependence of 5 Methyltetrahydrofolate Utilization by L5178Y Murine Leukemia Cells in vitro on the Presence of Hydroxycobalamin and Transcobalamin II. Cancer Res..

[2] Halpern B.C., Clark B.R., Hardy D.N., Halpern R.M., Smith R.A. (1974). The Effect of Replacement of Methionine by Homocystine on Survival of Malignant and Normal Adult Mammalian Cells in Culture. Proc. Natl. Acad. Sci. USA.

[3] Hoffman R.M., Erbe R.W. (1976). High in vivo Rates of Methionine Biosynthesis in Transformed Human and Malignant Rat Cells Auxotrophic for Methionine. Proc. Natl. Acad. Sci. USA.

[4] Judde J.G., Frost P. (1988). Patterns of Methionine Auxotrophy in Normal and Neoplastic Cells: The Methionine Independence of Lymphocyte Mitogenesis and Low Frequency of the Methionine-Dependent Phenotype in Human Tumors. Cancer Res..

[5] Guo H.-Y., Herrera H., Groce A., Hoffman R.M. (1993). Expression of the Biochemical Defect of Methionine Dependence in Fresh Patient Tumors in Primary Histoculture. Cancer Res..

[6] Fiskerstrand T., Christensen B., Tvsnes O.B., Ueland P.M., Refsum H. (1994). Development and Reversion of Methionine Dependence in a Human Glioma Cell Line: Relation to Homocysteine Remethylation and Cobalamin Status. Cancer Res..

[7] Zhang K., Xu P., Sowers J.L., Machuca D.F., Mirfattah B., Herring J., Tang H., Chen Y., Tian B., Brasier A.R. (2017). Proteome Analysis of Hypoxic Glioblastoma Cells Reveals Sequential Metabolic Adaptation of One-Carbon Metabolic Pathways. Mol. Cell. Proteom..

[8] Wang Z., Yip L.Y., Lee J.H.J., Wu Z., Chew H.Y., Chong P.K.W., Teo C.C., Ang H.Y.K., Peh K.L.E., Yuan J. (2019). Methionine Is a Metabolic Dependency of Tumor-Initiating Cells. Nat. Med..

[9] Klein S.G., Alsolami S.M., Steckbauer A., Arossa S., Parry A.J., Ramos Mandujano G., Alsayegh K., Izpisua Belmonte J.C., Li M., Duarte C.M. (2021). A Prevalent Neglect of Environmental Control in Mammalian Cell Culture Calls for Best Practices. Nat. Biomed. Eng..

[10] Mally J., Szalai G., Stone T.W. (1997). Changes in the Concentration of Amino Acids in Serum and Cerebrospinal Fluid of Patients with Parkinson’s Disease. J. Neurol. Sci..

[11] Hladky S.B., Barrand M.A. (2018). Elimination of Substances from the Brain Parenchyma: Efflux via Perivascular Pathways and via the Blood-Brain Barrier 11 Medical and Health Sciences 1109 Neurosciences. Fluids Barriers CNS.

[12] Cantor J.R., Abu-Remaileh M., Kanarek N., Freinkman E., Gao X., Louissaint A., Lewis C.A., Sabatini D.M. (2017). Physiologic Medium Rewires Cellular Metabolism and Reveals Uric Acid as an Endogenous Inhibitor of UMP Synthase. Cell.

[13] Hultberg B., Andersson A., Isaksson A. (1995). Metabolism of Homocysteine, Its Relation to the Other Cellular Thiols and Its Mechanism of Cell Damage in a Cell Culture Line (Human Histiocytic Cell Line U-937). BBA-Mol. Cell Res..

[14] Verbeek M.M., Leen W.G., Willemsen M.A., Slats D., Claassen J.A. (2015). Hourly Analysis of Cerebrospinal Fluid Glucose Shows Large Diurnal Fluctuations. J. Cereb. Blood Flow Metab..

[15] Muir A., Vander Heiden M.G. (2018). The Nutrient Environment Affects Therapy. Science.

[16] Vande Voorde J., Ackermann T., Pfetzer N., Sumpton D., Mackay G., Kalna G., Nixon C., Blyth K., Gottlieb E., Tardito S. (2019). Improving the Metabolic Fidelity of Cancer Models with a Physiological Cell Culture Medium. Sci. Adv..

[17] Ackermann T., Tardito S. (2019). Cell Culture Medium Formulation and Its Implications in Cancer Metabolism. Trends Cancer.

[18] Tardito S., Oudin A., Ahmed S.U., Fack F., Keunen O., Zheng L., Miletic H., Sakariassen P.Ø., Weinstock A., Wagner A. (2015). Glutamine Synthetase Activity Fuels Nucleotide Biosynthesis and Supports Growth of Glutamine-Restricted Glioblastoma. Nat. Cell Biol..

[19] DeBerardinis R.J., Mancuso A., Daikhin E., Nissim I., Yudkoff M., Wehrli S., Thompson C.B. (2007). Beyond Aerobic Glycolysis: Transformed Cells Can Engage in Glutamine Metabolism That Exceeds the Requirement for Protein and Nucleotide Synthesis. Proc. Natl. Acad. Sci. USA.

[20] Lilja A., Bergström K., Hartvig P., Spännare B., Halldin C., Lundqvist H., Långstrom B. (1985). Dynamic Study of Supratentorial Gliomas with L-Methyl-11C-Methionine and Positron Emission Tomography Anders. AJNR Am. J. Neuroradiol..

[21] Kaschten B., Stevenaert A., Sadzot B., Deprez M., Degueldre C., Del Fiore G., Luxen A., Reznik M. (1998). Preoperative Evaluation of 54 Gliomas by PET with Fluorine-18- Fluorodeoxyglucose and/or Carbon-11-Methionine. J. Nucl. Med..

[22] Kläsner B.D., Krause B.J., Beer A.J., Drzezga A. (2010). PET Imaging of Gliomas Using Novel Tracers: A Sleeping Beauty Waiting to Be Kissed. Expert Rev. Anticancer Ther..

[23] Nawashiro H., Otani N., Uozumi Y., Ooigawa H., Toyooka T., Suzuki T., Katoh H., Tsuzuki N., Ohnuki A., Shima K. (2005). High Expression of L-Type Amino Acid Transporter 1 in Infiltrating Glioma Cells. Brain Tumor Pathol..

[24] Nozaki S., Nakatani Y., Mawatari A., Hume W.E., Wada Y., Ishii A., Tanaka M., Tsuyuguchi N., Doi H., Watanabe Y. (2022). First-in-Human Assessment of the Novel LAT1 Targeting PET Probe 18F-FIMP. Biochem. Biophys. Res. Commun..

[25] Van Dijken B.R.J., Schuuring B., Jeltema H.R., van Laar P.J., Enting R.H., Dierckx R.A.J.O., Stormezand G.N., van der Hoorn A. (2022). Ventricle Contact May Be Associated with Higher 11C Methionine PET Uptake in Glioblastoma. Neuroradiology.

[26] Steed T.C., Treiber J.M., Taha B., Engin H.B., Carter H., Patel K.S., Dale A.M., Carter B.S., Chen C.C. (2020). Glioblastomas Located in Proximity to the Subventricular Zone (SVZ) Exhibited Enrichment of Gene Expression Profiles Associated with the Cancer Stem Cell State. J. Neurooncol..

[27] Yamaki T., Shibahra I., Matsuda K.I., Kanemura Y., Konta T., Kanamori M., Yamakawa M., Tominaga T., Sonoda Y. (2020). Relationships between Recurrence Patterns and Subventricular Zone Involvement or CD133 Expression in Glioblastoma. J. Neurooncol..

[28] Fontán-Lozano Á., Morcuende S., Davis-López de Carrizosa M.A., Benítez-Temiño B., Mejías R., Matarredona E.R. (2020). To Become or Not to Become Tumorigenic: Subventricular Zone Versus Hippocampal Neural Stem Cells. Front. Oncol..

[29] Bender K., Träger M., Wahner H., Onken J., Scheel M., Beck M., Ehret F., Budach V., Kaul D. (2021). What Is the Role of the Subventricular Zone in Radiotherapy of Glioblastoma Patients?. Radiother. Oncol..

[30] Brockman A.A., Mobley B.C., Ihrie R.A. (2021). Histological Studies of the Ventricular–Subventricular Zone as Neural Stem Cell and Glioma Stem Cell Niche. J. Histochem. Cytochem..

[31] Comas S., Luguera E., Molero J., Balaña C., Estival A., Castañer S., Carrato C., Hostalot C., Teixidor P., Villà S. (2021). Influence of Glioblastoma Contact with the Subventricular Zone on Survival and Recurrence Patterns. Clin. Transl. Oncol..

[32] Ripari L.B., Norton E.S., Bodoque-Villar R., Jeanneret S., Lara-Velazquez M., Carrano A., Zarco N., Vazquez-Ramos C.A., Quiñones-Hinojosa A., de la Rosa-Prieto C. (2021). Glioblastoma Proximity to the Lateral Ventricle Alters Neurogenic Cell Populations of the Subventricular Zone. Front. Oncol..

[33] Pala A., Reske S.N., Eberhardt N., Scheuerle A., König R., Schmitz B., Beer A.J., Wirtz C.R., Coburger J. (2019). Diagnostic Accuracy of Intraoperative Perfusion-Weighted MRI and 5-Aminolevulinic Acid in Relation to Contrast-Enhanced Intraoperative MRI and 11 C-Methionine Positron Emission Tomography in Resection of Glioblastoma: A Prospective Study. Neurosurg. Rev..

[34] Kim J.K., Jung T.Y., Jung S., Kim I.Y., Jang W.Y., Moon K.S., Kim S.K., Kim J.H., Lee K.H. (2021). Relationship between Tumor Cell Infiltration and 5-Aminolevulinic Acid Fluorescence Signals after Resection of MR-Enhancing Lesions and Its Prognostic Significance in Glioblastoma. Clin. Transl. Oncol..

[35] Shimizu K., Tamura K., Hara S., Inaji M., Tanaka Y., Kobayashi D., Sugawara T., Wakimoto H., Nariai T., Ishii K. (2022). Correlation of Intraoperative 5-ALA-Induced Fluorescence Intensity and Preoperative 11C-Methionine PET Uptake in Glioma Surgery. Cancers.

[36] Inoue A., Nishikawa M., Ohnishi T., Yano H., Kanemura Y., Ohtsuka Y., Ozaki S., Nakamura Y., Matsumoto S., Suehiro S. (2021). Prediction of Glioma Stemlike Cell Infiltration in the Non–Contrast-Enhancing Area by Quantitative Measurement of Lactate on Magnetic Resonance Spectroscopy in Glioblastoma. World Neurosurg..

[37] Yang C., Tian G., Dajac M., Doty A., Wang S., Lee J.H., Rahman M., Huang J., Reynolds B.A., Sarkisian M.R. (2022). Slow-Cycling Cells in Glioblastoma: A Specific Population in the Cellular Mosaic of Cancer Stem Cells. Cancers.

[38] Nakajo K., Uda T., Kawashima T., Terakawa Y., Ishibashi K., Tsuyuguchi N., Tanoue Y., Nagahama A., Uda H., Koh S. (2022). Maximum 11C-Methionine PET Uptake as a Prognostic Imaging Biomarker for Newly Diagnosed and Untreated Astrocytic Glioma. Sci. Rep..

[39] Maeda Y., Yamamoto Y., Norikane T., Mitamura K., Hatakeyama T., Miyake K., Nishiyama Y., Kudomi N. (2021). Fractal Analysis of 11C-Methionine PET in Patients with Newly Diagnosed Glioma. EJNMMI Phys..

[40] Sugimura T., Birnbaum S.M., Winitz M., Greenstein J.P. (1959). Quantitative Nutritional Studies with Water-Soluble, Chemically Defined Diets. VIII. The Forced Feeding of Diets Each Lacking in One Essential Amino Acid. Arch. Biochem. Biophys..

[41] Cavuoto P., Fenech M.F. (2012). A Review of Methionine Dependency and the Role of Methionine Restriction in Cancer Growth Control and Life-Span Extension. Cancer Treat. Rev..

[42] Chaturvedi S., Hoffman R.M., Bertino J.R. (2018). Exploiting Methionine Restriction for Cancer Treatment. Biochem. Pharmacol..

[43] Guéant J.L., Oussalah A., Zgheib R., Siblini Y., Hsu S.B., Namour F. (2020). Genetic, Epigenetic and Genomic Mechanisms of Methionine Dependency of Cancer and Tumor-Initiating Cells: What Could We Learn from Folate and Methionine Cycles. Biochimie.

[44] Wanders D., Hobson K., Ji X. (2020). Methionine Restriction and Cancer Biology. Nutrients.

[45] Goseki N., Yamazaki S., Shimojyu K., Kando F., Maruyama M., Endo M., Koike M., Takahashi H. (1995). Synergistic Effect of Methionine-depleting Total Parenteral Nutrition with 5-Fluorouracil on Human Gastric Cancer: A Randomized, Prospective Clinical Trial. Jpn. J. Cancer Res..

[46] Epner D.E., Morrow S., Wilcox M., Houghton J.L. (2002). Nutrient Intake and Nutritional Indexes in Adults with Metastatic Cancer on a Phase I Clinical Trial of Dietary Methionine Restriction. Nutr. Cancer.

[47] Thivat E., Farges M.C., Bacin F., D’Incan M., Mouret-Reynier M.A., Cellarier E., Madelmont J.C., Vasson M.P., Chollet P., Durando X. (2009). Phase II Trial of the Association of a Methionine-Free Diet with Cystemustine Therapy in Melanoma and Glioma. Anticancer Res..

[48] Tan Y., Zavala J.S., Xu M., Zavala J.J., Hoffman R.M. (1996). Serum Methionine Depletion without Side Effects by Methioninase in Metastatic Breast Cancer Patients. Anticancer Res..

[49] Gay F., Aguera K., Sénéchal K., Tainturier A., Berlier W., Maucort-Boulch D., Honnorat J., Horand F., Godfrin Y., Bourgeaux V. (2017). Methionine Tumor Starvation by Erythrocyte-Encapsulated Methionine Gamma-Lyase Activity Controlled with per Os Vitamin B6. Cancer Med..

[50] Kearse M.G., Wilusz J.E. (2017). Non-AUG Translation: A New Start for Protein Synthesis in Eukaryotes. Genes Dev..

[51] Lin M., Zhang X., Jia B., Guan S. (2018). Suppression of Glioblastoma Growth and Angiogenesis through Molecular Targeting of Methionine Aminopeptidase-2. J. Neurooncol..

[52] Li Q.Z., Zuo Z.W., Zhou Z.R., Ji Y. (2021). Polyamine Homeostasis-Based Strategies for Cancer: The Role of Combination Regimens. Eur. J. Pharmacol..

[53] Hansen L.J., Yang R., Roso K., Wang W., Chen L., Yang Q., Pirozzi C.J., He Y. (2022). MTAP Loss Correlates with an Immunosuppressive Profile in GBM and Its Substrate MTA Stimulates Alternative Macrophage Polarization. Sci. Rep..

[54] Avila M.A., García-Trevijano E.R., Lu S.C., Corrales F.J., Mato J.M. (2004). Methylthioadenosine. Int. J. Biochem. Cell Biol..

[55] Marjon K., Cameron M.J., Quang P., Clasquin M.F., Mandley E., Kunii K., McVay M., Choe S., Kernytsky A., Gross S. (2016). MTAP Deletions in Cancer Create Vulnerability to Targeting of the MAT2A/PRMT5/RIOK1 Axis. Cell Rep..

[56] Mavrakis K.J., Robert McDonald E., Schlabach M.R., Billy E., Hoffman G.R., DeWeck A., Ruddy D.A., Venkatesan K., Yu J., McAllister G. (2016). Disordered Methionine Metabolism in MTAP/CDKN2A-Deleted Cancers Leads to Dependence on PRMT5. Science.

[57] Kryukov G.V., Wilson F.H., Ruth J.R., Paulk J., Tsherniak A., Marlow S.E., Vazquez F., Weir B.A., Fitzgerald M.E., Tanaka M. (2016). MTAP Deletion Confers Enhanced Dependency on the PRMT5 Arginine Methyltransferase in Cancer Cells. Science.

[58] Sanderson S.M., Mikhael P.G., Ramesh V., Dai Z., Locasale J.W. (2019). Nutrient Availability Shapes Methionine Metabolism in P16/MTAP-Deleted Cells. Sci. Adv..

[59] Barekatain Y., Ackroyd J.J., Yan V.C., Khadka S., Wang L., Chen K.C., Poral A.H., Tran T., Georgiou D.K., Arthur K. (2021). Homozygous MTAP Deletion in Primary Human Glioblastoma Is Not Associated with Elevation of Methylthioadenosine. Nat. Commun..

[60] Locasale J.W., Melman T., Song S., Yang X., Swanson K.D., Cantley L.C., Wong E.T., Asara J.M. (2012). Metabolomics of Human Cerebrospinal Fluid Identifies Signatures of Malignant Glioma. Mol. Cell. Proteomics.

[61] Ulanovskaya O.A., Zuhl A.M., Cravatt B.F. (2013). NNMT Promotes Epigenetic Remodeling in Cancer by Creating a Metabolic Methylation Sink. Nat. Chem. Biol..

[62] Palanichamy K., Kanji S., Gordon N., Thirumoorthy K., Jacob J.R., Litzenberg K.T., Patel D., Chakravarti A. (2017). NNMT Silencing Activates Tumor Suppressor PP2A, Inactivates Oncogenic STKs, and Inhibits Tumor Forming Ability. Clin. Cancer Res..

[63] Eckert M.A., Coscia F., Chryplewicz A., Chang J.W., Hernandez K.M., Pan S., Tienda S.M., Nahotko D.A., Li G., Blaženović I. (2019). Proteomics Reveals NNMT as a Master Metabolic Regulator of Cancer-Associated Fibroblasts. Nature.

[64] Bowman R.L., Wang Q., Carro A., Verhaak R.G.W., Squatrito M. (2017). GlioVis Data Portal for Visualization and Analysis of Brain Tumor Expression Datasets. Neuro. Oncol..

[65] Matta E., Kiribayeva A., Khassenov B., Matkarimov B.T., Ishchenko A.A. (2020). Insight into DNA Substrate Specificity of PARP1-Catalysed DNA Poly(ADP-Ribosyl)Ation. Sci. Rep..

[66] Sim H.W., Galanis E., Khasraw M. (2022). PARP Inhibitors in Glioma: A Review of Therapeutic Opportunities. Cancers.

[67] Audrito V., Messana V.G., Deaglio S. (2020). NAMPT and NAPRT: Two Metabolic Enzymes with Key Roles in Inflammation. Front. Oncol..

[68] Kujundžić R.N., Prpić M., Đaković N., Dabelić N., Tomljanović M., Mojzeš A., Fröbe A., Trošelj K.G. (2021). Nicotinamide N-methyltransferase in Acquisition of Stem Cell Properties and Therapy Resistance in Cancer. Int. J. Mol. Sci..

[69] Lautrup S., Sinclair D.A., Mattson M.P., Fang E.F. (2019). NAD+ in Brain Aging and Neurodegenerative Disorders. Cell Metab..

[70] Bockwoldt M., Houry D., Niere M., Gossmann T.I., Reinartz I., Schug A., Ziegler M., Heiland I. (2019). Identification of Evolutionary and Kinetic Drivers of NAD-Dependent Signaling. Proc. Natl. Acad. Sci. USA.

[71] Sharma P., Xu J., Williams K., Easley M., Elder J.B., Lonser R., Lang F.F., Lapalombella R., Sampath D., Puduvalli V.K. (2022). Inhibition of Nicotinamide Phosphoribosyltransferase (NAMPT), the Rate-Limiting Enzyme of the Nicotinamide Adenine Dinucleotide (NAD) Salvage Pathway, to Target Glioma Heterogeneity through Mitochondrial Oxidative Stress. Neuro. Oncol..

[72] Adams S., Teo C., McDonald K.L., Zinger A., Bustamante S., Lim C.K., Sundaram G., Braidy N., Brew B.J., Guillemin G.J. (2014). Involvement of the Kynurenine Pathway in Human Glioma Pathophysiology. PLoS ONE.

[73] Panitz V., Končarević S., Sadik A., Friedel D., Bausbacher T., Trump S., Farztdinov V., Schulz S., Sievers P., Schmidt S. (2021). Tryptophan Metabolism Is Inversely Regulated in the Tumor and Blood of Patients with Glioblastoma. Theranostics.

[74] Guastella A.R., Michelhaugh S.K., Klinger N.V., Fadel H.A., Kiousis S., Ali-Fehmi R., Kupsky W.J., Juhász C., Mittal S. (2018). Investigation of the Aryl Hydrocarbon Receptor and the Intrinsic Tumoral Component of the Kynurenine Pathway of Tryptophan Metabolism in Primary Brain Tumors. J. Neurooncol..

[75] Zaragoza-Ojeda M., Apatiga-Vega E., Arenas-Huertero F. (2021). Role of Aryl Hydrocarbon Receptor in Central Nervous System Tumors: Biological and Therapeutic Implications (Review). Oncol. Lett..

[76] Jin U.H., Karki K., Cheng Y., Michelhaugh S.K., Mittal S., Safe S. (2019). The Aryl Hydrocarbon Receptor Is a Tumor Suppressor-like Gene in Glioblastoma. J. Biol. Chem..

[77] Herring J.L., Rogstad D.K., Sowers L.C. (2009). Enzymatic Methylation of DNA in Cultured Human Cells Studied by Stable Isotope Incorporation and Mass Spectrometry. Chem Res Toxicol..

[78] Robertson K.D., Jones P.A. (2000). DNA Methylation: Past, Present and Future Directions. Carcinogenesis.

[79] Weng J.Y., Salazar N. (2021). DNA Methylation Analysis Identifies Patterns in Progressive Glioma Grades to Predict Patient Survival. Int. J. Mol. Sci..

[80] Tornaletti S., Pfeifer G.P. (1995). Complete and Tissue-Independent Methylation of CpG Sites in the P53 Gene: Implications for Mutations in Human Cancers. Oncogene.

[81] Buchmuller B.C., Kosel B., Summerer D. (2020). Complete Profiling of Methyl-CpG-Binding Domains for Combinations of Cytosine Modifications at CpG Dinucleotides Reveals Differential Read-out in Normal and Rett-Associated States. Sci. Rep..

[82] Ohgaki H., Kleihues P. (2005). Epidemiology and Etiology of Gliomas. Acta Neuropathol..

[83] Sowers J.L., Johnson K.M., Conrad C., Patterson J.T., Sowers L.C. (2014). The Role of Inflammation in Brain Cancer. Adv Exp Med Biol..

[84] Zhang M., Yang D., Gold B. (2019). Origin of Mutations in Genes Associated with Human Glioblastoma Multiform Cancer: Random Polymerase Errors versus Deamination. Heliyon.

[85] Costa G., Barra V., Lentini L., Cilluffo D., Di Leonardo A. (2016). DNA Demethylation Caused By 5-Aza-2’-Deoxycytidine Induces Mitotic Alterations and Aneuploidy. Oncotarget.

[86] González B., Navarro-jim M., Gennaro A., Jansen S.M., Granada I., Perucho M., Alonso S. (2021). Somatic Hypomethylation of Pericentromeric SST1 Repeats And Tetraploidization in Human Colorectal Cancer Cells. Cancers.

[87] Kanai Y. (2022). Amino Acid Transporter LAT1 (SLC7A5) as a Molecular Target for Cancer Diagnosis and Therapeutics. Pharmacol. Ther..

[88] Zhen H., Kawai N., Okada M., Okubo S., Tamiya T., Zhang X., Liu W., Huo J., Fei Z. (2012). Relation of 4F2hc Expression to Pathological Grade Proliferation and Angiogenesis in Human Brain Gliomas. Chin. J. Clin. Oncol..

[89] Shen J., Song R., Hodges T.R., Heimberger A.B., Zhao H. (2018). Identification of Metabolites in Plasma for Predicting Survival in Glioblastoma. Mol. Carcinog..

[90] Phillips H.S., Kharbanda S., Chen R., Forrest W.F., Soriano R.H., Wu T.D., Misra A., Nigro J.M., Colman H., Soroceanu L. (2006). Molecular Subclasses of High-Grade Glioma Predict Prognosis, Delineate a Pattern of Disease Progression, and Resemble Stages in Neurogenesis. Cancer Cell.

[91] Segerman A., Niklasson M., Haglund C., Bergström T., Jarvius M., Xie Y., Westermark A., Sönmez D., Hermansson A., Kastemar M. (2016). Clonal Variation in Drug and Radiation Response among Glioma-Initiating Cells Is Linked to Proneural-Mesenchymal Transition. Cell Rep..

[92] Kling T., Ferrarese R., Ó hAilín D., Johansson P., Heiland D.H., Dai F., Vasilikos I., Weyerbrock A., Jörnsten R., Carro M.S. (2016). Integrative Modeling Reveals Annexin A2-Mediated Epigenetic Control of Mesenchymal Glioblastoma. EBioMedicine.

[93] Minniti G., Salvati M., Arcella A., Buttarelli F., D’Elia A., Lanzetta G., Esposito V., Scarpino S., Maurizi Enrici R., Giangaspero F. (2011). Correlation between O6-Methylguanine-DNA Methyltransferase and Survival in Elderly Patients with Glioblastoma Treated with Radiotherapy plus Concomitant and Adjuvant Temozolomide. J. Neurooncol..

[94] Binabaj M.M., Bahrami A., ShahidSales S., Joodi M., Joudi Mashhad M., Hassanian S.M., Anvari K., Avan A. (2018). The Prognostic Value of MGMT Promoter Methylation in Glioblastoma: A Meta-Analysis of Clinical Trials. J. Cell. Physiol..

[95] Paz M.F., Fraga M.F., Avila S., Guo M., Pollan M., Herman J.G., Esteller M. (2003). A Systematic Profile of DNA Methylation in Human Cancer Cell Lines. Cancer Res..

[96] Karpf A.R., Matsui S.I. (2005). Genetic Disruption of Cytosine DNA Methyltransferase Enzymes Induces Chromosomal Instability in Human Cancer Cells. Cancer Res..

[97] Barra V., Schillaci T., Lentini L., Costa G., Di Leonardo A. (2012). Bypass of Cell Cycle Arrest Induced by Transient DNMT1 Post-Transcriptional Silencing Triggers Aneuploidy in Human Cells. Cell Div..

[98] Bergo A., Strollo M., Gai M., Barbiero I., Stefanelli G., Sertic S., Gigli C.C., Di Cunto F., Kilstrup-Nielsen C., Landsberger N. (2015). Methyl-CpG Binding Protein 2 (MeCP2) Localizes at the Centrosome and Is Required for Proper Mitotic Spindle Organization. J. Biol. Chem..

[99] Roussel-Gervais A., Naciri I., Kirsh O., Kasprzyk L., Velasco G., Grillo G., Dubus P., Defossez P.A. (2017). Loss of the Methyl-CpG-Binding Protein ZBTB4 Alters Mitotic Checkpoint, Increases Aneuploidy, and Promotes Tumorigenesis. Cancer Res..

[100] Hervouet E., Debien E., Campion L., Charbord J., Menanteau J., Vallette F.M., Cartron P.F. (2009). Folate Supplementation Limits the Aggressiveness of Glioma via the Remethylation of DNA Repeats Element and Genes Governing Apoptosis and Proliferation. Clin. Cancer Res..

[101] Cartron P.F., Hervouet E., Debien E., Olivier C., Pouliquen D., Menanteau J., Loussouarn D., Martin S.A., Campone M., Vallette F.M. (2012). Folate Supplementation Limits the Tumourigenesis in Rodent Models of Gliomagenesis. Eur. J. Cancer.

[102] Jung J., Kim L.J.Y., Wang X., Sanvoranart T., Hubert C.G., Prager B.C., Wu Q., Wallace L.C., Jin X., Mack S.C. (2017). Nicotinamide Metabolism Regulates Glioblastoma Stem Cell Maintenance. JCI Insight.

